# Integrating emotional and psychological support into the end-stage renal disease pathway: a protocol for mixed methods research to identify patients’ lower-level support needs and how these can most effectively be addressed

**DOI:** 10.1186/s12882-016-0327-2

**Published:** 2016-08-02

**Authors:** Francesca Taylor, Celia Taylor, Jyoti Baharani, Johann Nicholas, Gill Combes

**Affiliations:** 1 Institute of Applied Health Research, College of Medical and Dental Sciences, University of Birmingham, Birmingham, B15 2TT UK; 2 Warwick Medical School, University of Warwick, Coventry, CV4 7AL UK; 3 Renal Unit, Birmingham Heartlands Hospital, Heart of England NHS Foundation Trust, Bordesley Green East, Birmingham, B9 5SS UK; 4 Renal Unit, New Cross Hospital, The Royal Wolverhampton NHS Trust, Wolverhampton Road, Wolverhampton, WV10 0QP UK

**Keywords:** Emotional, Psychological, Distress, End-stage renal disease, Mixed methods research, Integrated care

## Abstract

**Background:**

As a result of difficulties related to their illness, diagnosis and treatment, patients with end-stage renal disease experience significant emotional and psychological problems, which untreated can have considerable negative impact on their health and wellbeing. Despite evidence that patients desire improved support, management of their psychosocial problems, particularly at the lower-level, remains sub-optimal. There is limited understanding of the specific support that patients need and want, from whom, and when, and also a lack of data on what helps and hinders renal staff in identifying and responding to their patients’ support needs, and how barriers to doing so might be overcome. Through this research we therefore seek to determine what, when, and how, support for patients with lower-level emotional and psychological problems should be integrated into the end-stage renal disease pathway.

**Methods/Design:**

The research will involve two linked, multicentre studies, designed to identify and consider the perspectives of patients at five different stages of the end-stage renal disease pathway (Study 1), and renal staff working with them (Study 2). A convergent, parallel mixed methods design will be employed for both studies, with quantitative and qualitative data collected separately. For each study, the data sets will be analysed separately and the results then compared or combined using interpretive analysis. A further stage of synthesis will employ data-driven thematic analysis to identify: triangulation and frequency of themes across pathway stages; patterns and plausible explanations of effects.

**Discussion:**

There is an important need for this research given the high frequency of lower-level distress experienced by end-stage renal disease patients and lack of progress to date in integrating support for their lower-level psychosocial needs into the care pathway. Use of a mixed methods design across the two studies will generate a holistic patient and healthcare professional perspective that is more likely to identify viable solutions to enable implementation of timely and integrated care. Based on the research outputs, appropriate support interventions will be developed, implemented and evaluated in a linked follow-on study.

**Electronic supplementary material:**

The online version of this article (doi:10.1186/s12882-016-0327-2) contains supplementary material, which is available to authorized users.

## Background

### Burden of end stage renal disease

End-stage renal disease (ESRD) is when kidney function has deteriorated to such a poor level that without renal replacement therapy (RRT), dialysis or transplantation, death is probable within weeks or months. While dialysis and renal transplantation are life-saving treatments, they are also demanding and impact appreciably on the everyday lives of ESRD patients, often negatively affecting emotional and psychological wellbeing. Many patients find the transition to dialysis frightening and traumatic [1–3]. They can continue to experience periods of distress throughout their time on dialysis, due to the stress of treatment, loss of sexual function, altered body image, and decreased physical and cognitive functioning, as well as knock-on effects for employment, relationships and lifestyle [4–6]. Transplant patients experience many of the same stresses, along with fear of transplant failure and significant distress if a transplant does fail [7].

The prevalence of depression or anxiety in the ESRD population is around four times higher than in the general adult population [8–10]. No robust data exist on the prevalence of lower-level emotional and psychological problems, defined as difficulties in coping effectively with diagnosis, physical symptoms and treatment, which result in distress, poor emotional adjustment and reduced quality of life [11, 12]. Nonetheless, a recent study found that more than a third of dialysis patients experienced emotional difficulties [3]. Furthermore, untreated lower-level problems are associated with withdrawal from dialysis treatment [13, 14]; poor medication and diet compliance [15–17]; and, reduced ability to engage in pre-RRT education and treatment choice [3, 18, 19].

### Patient support

During the last 10 years several national policy documents have highlighted the importance of meeting renal patients’ emotional and psychological needs [20–22]. Support for patients’ psychosocial needs is integral to the recommended management of all long-term conditions [23]. Policy also emphasises that mental health should have parity with physical health and be integrated into care pathways [24, 25].

Despite this supportive policy framework and the evidence that patients with ESRD have significant needs, management of patients’ emotional and psychological difficulties, particularly at the lower-level, remains sub-optimal. Access to support is often restricted to patients with higher-level needs requiring psychiatric or psychological interventions. Patients want improved lower-level support, particularly in the areas of coping and adjustment [18, 26, 27], yet their needs tend to be ignored and frequently remain untreated [18, 26, 28].

Adjustment to ESRD has been described as a dynamic and constant process rather than an experience with an end point [29]. Across the ESRD pathway, patients are regularly faced with different emotional stressors and changes to their health. However, there appear to be certain ‘crisis points’ or ‘transitions’ that generate particularly strong stressors, such as first diagnosis of ESRD and initiation of dialysis [1, 2, 27]. This suggests there may be potential in exploring further whether there are key points in the pathway when support needs are greater and thus where it may be more effective to target support. There is a lack of studies though, that examine emotional and psychological responses across the ESRD trajectory over time.

It has also been argued in the context of chronic diseases in general, that very low or no emotional distress is not always desirable. In some circumstances such as receiving a diagnosis, feeling distress may be considered normal and the absence of distress maladaptive. Distress is more likely to be problematic if it continues over a long period. Therefore low or no distress should perhaps not be the singular outcome of any support interventions. An additional objective should be to impact positively on the process of adjustment, by helping patients to manage negative emotions, or to maintain positive affect in response to stressors [30], particularly since positive and negative affect can be independent [31].

Adjustment and coping can be fostered by renal staff helping patients release stressful emotion, develop coping skills, build healthy emotional responses and re-establish a balance in their life [32]. This first requires that staff recognise patients with lower-level emotional and psychological needs. The apparent high prevalence of untreated lower-level distress in patients with ESRD suggests there would be value in some form of systematic appraisal or screening being incorporated into the dialysis and transplantation pathways. Screening in itself will not lead to better health outcomes, but can help identify patients whose distress would otherwise remain undetected [33], and enable targeted provision of appropriate evidence-based support interventions.

Given the significant proportion of patients with ESRD who could potentially benefit from improved support, the most feasible interventions are likely to be low-cost and easy to incorporate into everyday clinical practice. There is a lack of evidence on interventions of this type used by patients with ESRD, although positive health impacts have been recorded in response to physical exercise [34–36] and coping and empowerment skills [37, 38]. More substantive evidence within the context of long-term conditions in general highlights the beneficial health and wellbeing outcomes of peer support [39]; mindfulness-based therapy [39–41]; and cognitive behaviour therapy (CBT)/computerised CBT [39, 42].

Additionally, interventions that involve patients providing clinicians with written information about their emotional needs in advance of consultation, or that encourage patients to ask more questions during consultations, have been found to promote discussion of emotional issues, improving patients’ wellbeing and reducing anxiety [43–45]. Findings from an earlier linked study suggested that two different types of low-cost intervention, designed to adjust consultant-patient communication, both have the potential to equip consultants with the cognitive and behavioural tools that enable discussion of emotional issues during routine out-patient consultations with ESRD patients [27]. The interventions were first, a Patient Issues sheet on which prior to their consultation, patients could indicate from the issues shown relating to their illness, those they would like to talk about. Second, consultants asking patients a question adapted from the Patient Health Questionnaire (PHQ-9) [46] recommended by the National Institute for Health and Care Excellence [47], about whether they had been feeling down or miserable in the last week.

### Staff attitudes and behaviour

The reasons why renal staff do not identify or respond to the lower-level emotional and psychological needs of their patients with ESRD have not been well researched. There is some evidence that staff find it hard to recognise patients’ distress [26, 45], while cancer studies suggest many patients tend not to spontaneously express emotional concerns in clinics [44, 48]. Some patients may feel unable to disclose their feelings to staff who care for them regularly [49]. Cultural and ethnic background may also play a critical role in multicultural patient populations [50]. Additionally, it seems that doctors are reluctant to raise emotional issues during consultations, preferring to focus on the biomedical [51, 52]. Discussions with local renal consultants identified further barriers, including worries about lengthening consultation times, being unable to deal with the issues raised by patients, uncertainty about where to refer patients for support, and added costs.

Nurses and healthcare assistants (HCAs) are particularly well placed to support ESRD patients as they spend more time and develop closer relationships with patients, often over many years. There is an absence of evidence, however, about what prevents them from addressing patients’ psychosocial needs. Notably, though, there is relevant research conducted in other long-term conditions to indicate that clinicians and health staff in general can be educated and trained to effectively deliver support to patients in distress [53, 54].

### Local context

Several local NHS Trusts have expressed interest in implementing low-cost interventions to better support the psychosocial needs of their ESRD patients. However, renal clinicians from the Trusts recognise that to facilitate implementation it will be important to accurately target appropriate interventions towards those patients for whom the need is greatest, and when and where in the ESRD pathway effect is likely to be strongest. Additionally, it is acknowledged that existing barriers to renal staff identifying and responding to patient needs will have to be overcome.

### Aims and objectives

While there is robust evidence that ESRD patients have emotional and psychological needs, there is less data on the specific support that patients need and want, from whom, and at which points in the ESRD pathway. We also have limited knowledge of what helps or hinders renal staff in identifying and responding to their patients’ needs. Therefore, the overarching aim of the research is to determine what, when and how support for patients with lower-level emotional and psychological needs should be integrated into the ESRD pathway. The research will involve two linked studies.

The aim of Study 1 is to identify what support is needed and wanted, when, and by which ESRD patients with lower-level emotional and psychological needs. The objectives are to:

- Determine whether there are quantifiable differences in the levels of distress experienced by patients at different points in the ESRD pathway
- Explore ESRD patients’ needs, wants and expectations of support for lower-level emotional and psychological difficulties
- Determine whether there are patient groups who need and want more support than others for lower-level emotional and psychological difficulties
- Explore whether there are certain points along the ESRD pathway when patients should be screened for emotional distress and offered support interventions
- Assess which interventions could potentially help most, for whom, and under which circumstances.

For Study 2, the aim is to assess what helps or hinders renal staff in identifying and responding to ESRD patients with lower-level emotional and psychological needs, and how barriers to doing so might be overcome. The objectives are to:

- Explore staff views about what support is required for patients at key points in the ESRD pathway and when this support might be needed most
- Explore what staff think are the components of good support for lower-level emotional and psychological needs and whose role it is to meet these needs
- Detail how patients with lower-level emotional and psychological needs are currently identified, how these are supported and by whom
- Determine what factors help or hinder staff in identifying and responding to patients’ lower-level emotional and psychological needs
- Explore what interventions, tools, resources, training and support are needed to improve how staff identify and respond to patients’ lower-level emotional and psychological needs
- Identify and explain differences and similarities between sites, including how local context influences practice.

The intelligence collected from the two studies will be used to develop a theoretical model for collaborative care management in ESRD that can aid local renal staff in designing and targeting appropriate low-cost, evidence-based interventions to support patients’ lower-level emotional and psychological needs. Based on this model, local staff will develop appropriate interventions for implementation and evaluation in a planned follow-on study. The results from Studies 1 and 2 will also provide a baseline against which future patient and staff views can be assessed after implementation of the interventions.

## Methods/Design

### Funding and governance

The research is funded by the National Institute for Health Research (NIHR) programme, Collaborations for Leadership in Applied Health Research and Care West Midlands (CLAHRC WM). The conduct of the research will be overseen by an Advisory Group which will meet at 4-monthly intervals throughout the research programme and includes renal clinicians, psychologists, academics, the Principal Investigators (PIs), and a home haemodialysis (HHD) patient.

### Setting

Studies 1 and 2 will be undertaken in two Renal Centres in the West Midlands. The sites recruited into the study have variable ESRD patient populations (see Table 1); psychological support services; current interventions to support ESRD patients with lower-level emotional and psychological needs and organisation of dialysis and transplant services.

**Table 1:** Numbers of patients at each stage of ESRD pathway

	Study site 1	Study site 2	Total	40 % response rate
Pre-dialysis	180^a^	180^a^	360	144
On dialysis less than 2 years, not on transplant list	114	111	225	90
On dialysis more than 2 years, not on transplant list	213	259	472	189
On dialysis and on transplant list	56^c^	106^c^	162	65
With transplant for any time period	180^b^	182^b^	362	145
Total all stages	743	838	1581	633

*Sources*: Estimates based on figures from Renal Registry, 2014 (Tables 1.4, 5.9, 5.11); ^a^Trust figures, September 2015; ^b^Renal Registry, 2014 (Table 2.2.); ^c^ Estimates based on figures from Renal Registry, 2013 (Tables 2.2 and 4.2) and Renal Registry, 2014 (Tables 1.4 and 2.2)

### Overview of design

A convergent, parallel mixed methods design [55] will be employed for both studies. Mixed research methods (combined qualitative and quantitative) have been chosen because of the key benefits of triangulation: providing context, illustration, completeness and credibility [56]. A mixed methods design is also particularly useful in the development and subsequent evaluation of a complex intervention [57]. Qualitative and quantitative data will be collected separately. For each study, both data sets will be analysed separately and the results then compared or combined using interpretive analysis.

A further stage of synthesis will use data-driven thematic analysis [58] to describe and interpret findings, looking for: triangulation of themes from multiple data sources; frequency of themes across pathway stages; patterns and plausible explanations of observed effects. We will also explore generalisability through a more in-depth exploration of the qualitative data, as used by Gillard et al. [59], to consider whether findings reflect localised issues that are context-specific, or whether they are likely to apply to all ESRD pathways.

### Participants

Patients at five different points across the ESRD pathway, and renal staff working with them, will be studied. Existing evidence, and discussion with clinicians in the study sites, indicates that ESRD patients are likely to experience different types and levels of distress at five key stages of the pathway. The five stages are (1) diagnosed with chronic kidney disease (CKD) stage 5 and still pre-RRT; (2) on dialysis (peritoneal dialysis [PD], haemodialysis [HD], or HHD) for less than 2 years, and not on the transplant list; (3) on dialysis for more than 2 years, and not on the transplant list; (4) on dialysis and on the transplant list; and (5) with functioning transplant.

### Literature review

A summary literature review will be conducted using social science based literature published in the UK and internationally from studies undertaken among patients with ESRD and renal staff who work with them. Studies will also be included that have involved patients with other comparable long term conditions and at similar disease stages (advanced/severe/end stage/incurable), and staff who work with them: for example, end-stage chronic obstructive pulmonary disease, advanced cancer, chronic disease at palliative end of life care.

Publications will be surveyed from 1995 to 2015. Searches will be undertaken of the following databases: MEDLINE, Embase, PsychINFO, CINAHL, Cochrane Library, Web of Science.

The literature review will address these specific research questions:

1. Which interventions to support lower-level emotional and psychological needs are likely to help most, and for whom, under which circumstances?
2. Are there any generic low-cost interventions that might be adopted for use with all ESRD patients?
3. Are there interventions that can provide support for renal staff negatively affected by supporting the emotional needs of ESRD patients?

### Patient and public involvement

The research design has been driven from the start by renal patients’ needs. The idea for the research study came from listening to ESRD patients at a half-day Workshop held in June 2014 attended by 32 patients and renal clinicians. The Workshop aims were to present and reflect on findings from an earlier linked study of two interventions designed to prompt discussion of emotional issues during outpatient consultations [27], and brainstorm ideas for future research. The priority research areas identified by patients were to explore: which interventions to support emotional needs best suit which types of ESRD patient; how renal staff can be encouraged and enabled to support patients’ emotional needs.

Several patients attending the Workshop volunteered to be members of a renal patient and public involvement (PPI) reference group. They have been closely involved in the design of this research and will remain actively involved throughout the work, contributing their perspectives on emerging and final research findings and how these should be disseminated and implemented. In addition, the researchers will continue to work closely with a CLAHRC PPI Forum that meets every three months. The four PPI Forum advisors have contributed to the design of the study questionnaires and interview topic guides, and will review and disseminate study findings. This process will ensure the research outputs have relevance and benefit to patients.

## Study 1: patients

### Participant inclusion and exclusion criteria

#### Inclusion

1. Adult patients aged 18 years and over, receiving treatment at one of the study sites, with a diagnosis of CKD stage 5 and at one of the identified five key stages of the ESRD pathway.
2. Clinically stable and well enough to take part.
3. Have the capacity to give informed consent.
4. Willingness to take part.
5. Not had contact with psychiatric services (including seeing a psychiatrist) since diagnosis with CKD stage 5.

#### Exclusion

1. Lacking capacity to give informed consent.
2. Had contact with psychiatric services (including seeing a psychiatrist) since diagnosis with CKD stage 5.

### Quantitative data

#### Sampling

Patients who meet the inclusion criteria will be grouped according to the five identified stages of the ESRD pathway. Estimated numbers of eligible patients at each of these stages in the two study sites are shown in Table 1. Assuming a questionnaire response rate of 40 %, based on previous patient surveys in these sites, we could achieve the sample sizes shown in the last column. Given the relatively low patient numbers at two of the stages, we propose inviting all patients who meet the study inclusion criteria to complete the questionnaire in the study sites (743 patients in site 1, 838 patients in site 2).

#### Recruitment

A Renal Unit staff member in each study site will identify all eligible ESRD patients from patient lists. These patients will be sent a questionnaire ‘pack’ by the lead consultant of their Renal Unit, containing a letter about the study, a hard copy questionnaire and pre-paid reply envelope addressed to a study researcher at the University. Additionally, the pack will include a Study Information Sheet, outlining the study purpose and what participation will involve, with contact details for a study researcher if there are any queries about the study, and for nurse support if a patient participant is feeling distressed or upset. The Information Sheet will also suggest that patients with language difficulties ask someone they know to help them complete the questionnaire.

To help maximise the response rate, patients that have not returned the questionnaire after three weeks will be sent another questionnaire pack. In order to identify these patients while also maintaining patient anonymity, the study researchers will mark each envelope containing the questionnaire pack with a different number, plus an initial indicating the study site. They will mark the same number and initial on the enclosed reply-paid envelope. Each study site will be sent the appropriate questionnaire packs for despatch. The renal staff member in each site assigned to addressing and despatching the packs to eligible patients, will then record the relevant number on their patient list. Three weeks after the questionnaire packs have been sent to patients, the study researchers will collate all the numbers on returned envelopes and then inform the relevant staff member of the returned numbers for their site. The staff members will then know, by comparing the returned numbers to the numbers recorded on their patient list, which patients have not returned a completed questionnaire and therefore should be sent a second pack.

#### Data collection

All patients in the study sample will be invited to complete a self-report questionnaire (Additional file 1). The questionnaire will include validated measures for assessing different aspects of emotional distress, and adjustment to emotional stressors, in combination with additional questions about emotional support and intervention use. We have deliberately chosen short validated measures, which together with the additional questions, should not take longer than 20 min to complete and avoid over-burdening patients.

#### Measures employed in the study and rationale

- ***Validated measures***

The Distress Thermometer (DT) [60] has been selected as the primary measure of distress for this study; and will be used to enable comparisons of the level of distress at different stages in the ESRD pathway and how support needs vary at different levels of distress. There are few validated tools for measuring emotional and psychological needs at the lower-level among ESRD patients, or chronic disease patients in general. Existing studies have tended to use validated tools designed to measure anxiety and depression e.g. the Hospital Anxiety and Depression Score (HADS), the Beck Depression Inventory (BDI, BDI-1A, BDI-11), looking for scores below the thresholds set for detecting the clinical presence of anxiety or depression. An advantage of these tools is that they are reliable measures, validated for use with renal patients. However, they appear to restrict lower-level emotional issues to those linked with low-level anxiety and depression, and therefore, are not suitable for this study which uses a broader definition, encompassing a range of symptoms, emotions and experiences associated more with distress (see Background).

There are two main tools which have been developed to measure distress: the Brief Symptom Inventory (BSI) and the more recently designed DT. The DT is a measure of global distress initially developed by Roth et al. [60] for the National Comprehensive Cancer Network (NCCN). The term ‘distress’ was chosen by NCCN because it is “more acceptable and less stigmatizing than ‘psychiatric’, ‘psychosocial’ or ‘emotional’, sounds ‘normal’ and less embarrassing, and can be measured by self-report” [61]. Although developed for cancer patients, the DT has been validated for use in a range of chronic disease populations, including more recently a UK renal population [62]. Furthermore, being a visual measure makes the DT particularly helpful for people with language difficulties [39].

In cancer populations, a score of 4 or 5 (on a 0–10 scale) on the DT is defined as mild distress, a score of 6 or 7 as moderate distress, and 8 or higher as severe distress [63]. For this study, we will classify ESRD patients who score between 4 and 7 on the DT as having lower-level emotional and psychological problems. A problem list accompanies the DT to identify causes for distress and this will also be included in the study questionnaire.

We will use the DT contained within the Emotion Thermometers (ET) [63, 64]. The ET is a simple five-domain visual analogue scale. Each domain is self-rated on an 11-point (0 to 10) Likert scale. In addition to the Distress Thermometer, it contains the Anxiety, Depression, Anger, and Help Thermometers. Patients score these thermometers on the basis of how much emotional upset they have experienced during the preceding week, including the present day. Containing a combination of items, the ET has been found with cancer patients to be more accurate than a single domain for the identification of any significant emotional difficulty, as well as the assessment of broadly defined distress; 51 % of patients who scored below the cut-off point for distress using the DT recorded emotional difficulties on the ET [64]. Furthermore, the ET is very quick to complete (less than one minute) with non-completion rates low in comparison with conventional measures [63, 64].

The Positive and Negative Affect Schedule [PANAS] will be used to measure patients’ adjustment to emotional stressors. Developed and validated by Watson et al. [32], this 20-item instrument that measures positive and negative affect has been widely used in different chronic disease populations and some renal populations [65]. Positive Affect (PA) reflects the extent to which a patient feels enthusiastic, active and alert, while the Negative Affect (NA) dimension assesses subjective distress and discomfort. PA and NA have high internal consistency and are largely uncorrelated [32].

Each item of the PANAS is rated on a 5-point scale to indicate the extent to which the respondent has felt this way in general or a specific time-period such as ‘in the past few hours’ or ‘during the past week’. We will use a time period of ‘during the last week’.

b) ***Additional measures***

Patients’ perceptions and views will also be measured on: recent events that have caused distress; ability to cope with their illness and treatment; extent to which feel supported by renal staff; interventions used for distress; benefits and satisfaction from receiving support for distress from renal staff; practical ideas about future support for distress.

Most of these questions will have pre-set answer options using an 11-point (0–10) Likert response scale [66]. Negatively and positively worded answer options will be balanced to avoid ‘yea’ saying’. An open-ended question at the end will allow free-text responses, to avoid missing any types of support considered important through use of pre-set answer options. Tick-box data will be collected on age, gender, ethnicity, relationship status, length of time since diagnosis, length of time on dialysis or with a functioning renal transplant, and dialysis treatment type. Patients will also be asked whether they received any assistance in completing the questionnaire. Additionally, respondents will be asked if they are willing to participate in a further individual telephone or face-to-face interview, and if so to provide their contact details.

#### Sample size calculation

The sample size calculation – based on the study objective to determine whether there are any differences in distress at different stages of the ESRD pathway - assumes a 1.5-point difference on the DT between patient groups (pathway stages) with the largest and smallest means. A two-sided alpha of 0.05 and 90 % power have been used. This gives an effect size, f, of 0.20. A sample size calculation (using G*Power version 3.1.9.2) gives a total sample size of 485, approximately 97 in each patient group across the two study sites. Since group sizes will not be equal, ideally this would be the sample size requirement for the smallest group, although it is noted that this may not be met in practice for all groups (see Table 1).

#### Analysis

One-way ANOVA will be used to analyse data from the DT, ET, and PANAS, combining data across both sites. If there is a statistical significant difference on the main analysis measure, the DT, between patients at different stages on the ESRD pathway, then appropriate post-hoc t-tests to compare individual patient groups will be undertaken, with *p*-values corrected for multiple comparisons using the Bonferroni Adjustment. Comparisons of scores between patient groups and study sites will be made using two-way ANOVA, although we may not have sufficient power to detect small differences between sites.

The remaining questionnaire data, although on Likert scales, will initially be analysed as continuous data, using means and standard deviations or medians and inter-quartile ranges as appropriate for the distribution. Scores from individual items in each question-group will be summed prior to analysis e.g. to give a composite ‘coping’ score. Appropriate corrections for multiple comparisons will be made given that multiple questions are being asked.

### Qualitative data

#### Sampling

Sampling will be purposive, based on the study inclusion criteria and to provide maximum diversity of age, gender, ethnicity, and stage in the ESRD pathway. The aim is to conduct 25 interviews - five interviews with patients at each of the five stages of the ESRD pathway - in each of the two study sites, or until thematic saturation is achieved. Refusal rates have varied widely, between 30 and 69 %, for recent studies in ESRD patient populations undertaken by the study researchers in the study sites [3, 29]. Therefore, to achieve this sample size, an initial sample of 85 patients – 17 patients at each of the five stages of the ESRD pathway - will be drawn up in each site.

#### Recruitment

This will be undertaken from amongst two patient groups. First, patients who have completed the questionnaire, scored between 4 and 7 on the DT, and expressed a willingness to participate in a telephone or face-to-face interview. Second, patients who have completed the questionnaire, scored less than 4 on the DT but between 4 and 7 on any of the other Emotion Thermometers, and expressed a willingness to participate in an interview.

A study researcher will send these patients a Consent Form, together with a Study Information Sheet outlining the study purpose and what participation will involve. It will also include the same contact details as provided in the questionnaire Information Sheet. Patients would be contacted by phone at least 7 days later by a study researcher, and a suitable date and time arranged for interview with those who consent to participate. Translation and interpretation facilities will be made available for participants who may have difficulties in adequately understanding written and verbal information in English. For all patients, written consent will be taken by a study researcher prior to the start of the interview.

#### Data collection

The interviews will be in-depth and semi-structured, allowing key issues to be explored without being overly prescriptive about content and direction. An interview topic guide has been developed to cover the following areas:

- Experience of emotional difficulties and needs linked to their illness and/or treatment, when and for how long.
- Language used around emotional difficulties and needs, and its meaning.
- Whether and how emotional needs have been recognised and supported by renal staff, when, and by whom.
- What, if any, support used, when and why.
- Likes and dislikes of support used.
- Support would have liked/would want in future, when, and from whom.
- Key elements would want included in an emotional support intervention.

Individual face-to-face interviews will be conducted in each patient’s Hospital Trust, in a quiet room away from the Renal Unit, where confidentiality is assured. Telephone interviews will be conducted in a sound-proof room on University premises. The interviews will last 35–45 min and be audio-recorded. Field notes will be taken to record key thoughts and issues. Audio-recordings will be professionally transcribed in full and transcripts proof-read against the recordings.

#### Analysis

The interviews will be analysed using thematic analysis [67]. The analysis will be guided by the overall aims and objectives of the study, supplemented by the researchers’ identification of themes based on the views and experiences of patients. At least two researchers will independently analyse and code transcripts from a sub-sample of a minimum of a third of the interviews. The results will be compared, discussed and reviewed until agreement is reached. An initial framework of themes will then be developed, together with a code book, and used to structure verbatim responses onto a spreadsheet. The codes and themes will be refined and elaborated collectively as more data is collected. As sequential analysis progresses, significant data will be compressed so as to adhere around key analytic themes. Where the data collected does not fit into existing themes, new themes will be developed or existing ones revised until all the data is coded by theme.

## Study 2: renal staff

### Participant inclusion and exclusion criteria

#### Inclusion

1. Staff currently working with patients at one of the five stages of the ESRD pathway, or staff with a renal managerial role.
2. Employed by the hospital/sub-contractor at one of the study sites for a minimum of two months.

#### Exclusion

1. Agency or bank staff.

### Design and framework

The study draws on theoretical perspectives linked to the implementation of change within healthcare. This is relevant because staff report difficulties in implementing long-standing national guidance which recommends the provision of emotional and psychological support for patients with ESRD. In particular, we have focused on the research of Oxman [68], Ferlie and Shortell [69], and Grol and Grimshaw [70], who have similar frameworks for analysing factors that help or hinder the implementation of change. Our analytical framework will identify factors operating at three levels: the individual staff member (e.g. skills or confidence); the team or Renal Unit (e.g. time or training); and the organisation or Hospital Trust as a whole (e.g. resources). This framework will enable us to develop a detailed understanding of what helps or hinders staff in identifying and responding to patients with lower-level emotional and psychological difficulties and insight into what tools, resources, training and support are effective, and for which staff, to help improve support for patients.

### Quantitative data

#### Sampling

All staff in the two participating study sites who meet the inclusion criteria will be invited to complete the online questionnaire: site 1–165 staff; site 2–188 staff. On the basis of previous staff surveys in these sites, we anticipate around a 70 % response rate. This would result in approximately 247 returns (115 site 1; 132 site 2).

#### Recruitment

Staff will be sent an email about the study by their site clinical lead, with a Study Information Sheet explaining the study purpose, why they are being asked to take part in the study, and what participation will involve. It will also provide contact details for a study researcher if there are any queries about the study, and for the Trust Counselling Service, if a staff participant is feeling distressed or upset. The email will contain a direct web-link to access the questionnaire online. If three weeks after the initial email to staff, response rates are lower than the anticipated 70 %, a reminder e-mail will be sent.

#### Data collection

The questionnaire (Additional file 2) will survey staff views about the following issues in relation to ESRD patients with lower-level emotional and psychological needs:

- Perception of the proportion of all ESRD patients in their Renal Unit with lower-level needs.
- Perceived benefits and difficulties of identifying and responding to patients’ needs.
- Level of satisfaction with how patients’ needs are currently met.
- Extent to which the identification and response to patients’ needs is seen as part of current role, and who else has responsibility for this in the Renal Unit (‘role’).
- How skilled, confident and well trained they feel to identify and respond to patients’ needs (‘capacity’).
- Practical ideas about what could help in identifying and responding to patients’ needs in future.

The two dimensions of ‘role’ and ‘capacity’ have been selected as the primary outcome measures for this study. Existing evidence suggests that these two dimensions are likely to encompass the key issues which help or hinder renal staff in identifying and responding to the lower-level emotional and psychological needs of ESRD patients (see Background – Staff attitudes and behaviour).

A self-completion questionnaire will be used for ease of response, taking about 10 min to complete, with most questions having pre-set answer options using an 11-point (0–10) Likert response scale [66]. Negatively and positively worded answer options will be balanced to avoid ‘yea-saying’. Open-ended questions at the end will allow free-text responses in order to avoid missing issues through the use of pre-set answer options. Tick-box data will be collected for respondents’ age, gender, role, frequency of ESRD patient contact, and length of time in post and since qualification. Additionally, respondents will be asked if they are willing to participate in a further individual telephone or face-to-face interview, and if so to provide their contact details.

#### Analysis

The data will be analysed as continuous data, using means and standard deviations or medians and inter-quartile ranges as appropriate for the distribution. Scores from individual items in each question-group will be summed prior to analysis e.g. to give composite ‘role’ and ‘capacity’ scores.

### Qualitative data

#### Sampling

Purposive sampling will select interviewees from staff completing the questionnaire who have expressed a willingness to participate in a telephone or face-to-face interview, and based on: role, gender, time in current post and since qualification. An initial sample of 22 staff per site will be drawn up, with interviews conducted until saturation is achieved, provided all staff groups have been included. This is expected to be achieved with 18–20 staff per site, based on previous qualitative staff research in the study sites, where the refusal to participate rate was only 13 % [3]. We therefore expect to have a total sample size of 36–40 staff. Each site’s sample is expected to include: 3 consultants; 1 registrar; 6 nurses and 6 HCAs selected from clinical areas: pre-dialysis, transplant, unit HD, HHD, PD, wards; 1 dietician; 1 technician; 1 psychologist/counsellor/social worker/welfare advisor; 1 Renal Unit manager. Precise numbers in each category may vary between sites. The majority of the sample will be made-up of nurses and HCAs, since these staff members have most contact with patients, and an in-depth understanding is needed about why they are not identifying and responding to their patients’ emotional and psychological needs.

#### Recruitment

Staff that have completed the questionnaire and expressed a willingness to participate in a telephone or face-to-face interview will be sent a Consent Form and Study Information Sheet by a study researcher. The Information Sheet will explain the purpose of the interview, and what participation will involve, and include the same contact details as provided in the questionnaire Information Sheet. Staff would be contacted by phone at least 7 days later by a study researcher, and a suitable date and time arranged for interview with those who consent to participate.

In addition, staff that have completed the questionnaire but not answered the question about being willing to participate in a telephone or face-to-face interview will be sent an email by the lead consultant of their Renal Unit, along with a Consent Form and Study Information Sheet. The email will explain that a designated member of staff from their Renal will contact them the following week to ask whether they were interested in participating in an individual telephone or face-to-face interview, and if willing, a suitable date and time for interview arranged. Contact details of staff willing to participate in the study would then be provided to the study researchers.

#### Data collection and analysis

The interviews will be in-depth and semi-structured, and conducted in the same locations employed for the Study 1 patient interviews. They will be used to explore and seek explanations of staff attitudes, perceptions and perspectives; to determine how barriers to staff identifying and responding to the emotional needs of ESRD patients can be overcome; and, how appropriate changes can be implemented. An interview topic guide has been developed to cover the following areas in relation to ESRD patients with lower-level emotional and psychological needs:

- How are patients’ needs identified and supported (by individual, team/Renal Unit, organisation/Hospital Trust)
- Perceptions of the individual/team-Renal Unit/Hospital role in identifying and responding to patients’ needs
- What factors currently help or hinder identification and support of patients’ needs.
- Views on the components of good lower-level emotional and psychological support.
- How skilled, confident and well trained do they feel to identify and support patients.
- What needs to change or improve to enable better identification and support of patients (interventions, tools, resources, training, support).
- Views on how these changes or improvements can best be facilitated and effectively implemented.

The data collected will be analysed using the same methodological approach as the Study 1 patient interviews.

## Discussion

This is important and challenging research since the management of lower-level emotional and psychological difficulties experienced by patients with ESRD remains sub-optimal, despite patients’ desire for improved psychosocial support and an encouraging policy background. However, the use of a mixed methods approach across the two research studies, incorporating qualitative and quantitative components, will generate a holistic perspective that is more likely to identify viable solutions, enabling integrated care for patients to be incorporated into the ESRD pathway. By considering the perspectives of both the ESRD patient and the healthcare professional, it should be possible to develop interventions that are useful to patients, but feasible to implement when taking into account professional and organisational barriers. Based on the research outputs, appropriate interventions to support ESRD patients with lower-level emotional and psychological needs will be developed, implemented and evaluated in a linked follow-on study. We aim to disseminate the research findings through PPI groups involved in the work, renal peer-reviewed scientific journals, local patient and clinician workshops, and national conferences.

## Abbreviations

BDI, beck depression inventory; BDI-11, beck depression inventory – version 11; BDI-1A, beck depression inventory – version 1A; BSI, brief symptom inventory; CBT, cognitive behaviour therapy; CI, Chief Investigator; CKD, chronic kidney disease; CLAHRC WM, Collaborations for Leadership in Applied Health Research and Care West Midlands; DT, depression thermometer; ESRD, end-stage renal disease; ET, emotion thermometers; HADS, hospital anxiety and depression score; HCA, healthcare assistant; HD, haemodialysis; HHD, home haemodialysis; NA, negative affect; NCCN, National Comprehensive Cancer Network; NIHR, National Institute for Health Research; PA, positive affect; PANAS, positive and negative affect schedule; PD, peritoneal dialysis; PI, principal investigator; PPI, patient and public involvement; RCT, randomised controlled trial; RRT, renal replacement therapy

## Additional files

Additional file 1: Patient questionnaire. Questionnaire developed for Study 1. (PDF 1007 kb) Additional file 2: Staff questionnaire. Questionnaire developed for Study 2. (PDF 2781 kb)
