# Supplementary material for: Integrating emotional and psychological support into the end-stage renal disease pathway: a protocol for mixed methods research to identify patients’ lower-level support needs and how these can most effectively be addressed
Source: BMC Nephrol. 2016 Aug 2;17:111. doi: 10.1186/s12882-016-0327-2 (PMC4971672; doi:10.1186/s12882-016-0327-2)
Supplement: Additional file 1: — Patient questionnaire. Questionnaire developed for Study 1. (PDF 1007 kb) [file 12882_2016_327_MOESM1_ESM.pdf]

UNIVERSITY OF BIRMINGHAM

Supporting the Emotional Needs of Renal Patients

Patient Questionnaire

We are inviting you to take part in this study on supporting the emotional needs of renal patients. We are interested in your views. The information collected will help improve future renal support services.

If you agree to take part, please fill-in this questionnaire.
The answers you provide will be kept strictly confidential.

Who should complete the questionnaire?

The questions should be answered by the person named on the front of the envelope. If you need help to complete the questionnaire, the answers should be given from **your** point of view – not the point of view of the person helping you to fill it in.

Completing the questionnaire

Please use a black or blue pen to complete the questionnaire. For each question where there are circle/s to fill in, please fill in the circle that is closest to your views.

Please fill in circles like this: ● **Not like this: ⊗ or** 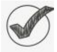

Don't worry if you make a mistake: simply cross out the mistake and fill in the correct circle.

Many of the questions relate to *distress*. By *distress* we mean the unpleasant feelings or emotions that may interfere with your ability to cope with kidney failure, its physical symptoms and its treatment. Distress covers a wide range of feelings including anger, frustration, sadness, fear, depression, guilt and anxiety.

We encourage you to answer all the questions. However, if there is a question you do not want to answer, you can choose to leave it blank, and just go onto the next question.

Questions or help?

If you have any queries about the questionnaire, or need support, please call one of the telephone numbers given in the letter enclosed with this questionnaire.

These first questions are about your feelings and emotions, and what influences them.

Q. 1.

<u>Instructions</u>
In the first four columns, please ***circle the number*** *(0-10) that best describes how much emotional upset you have been experiencing in the past week, including today.*
In the last column, please indicate how much help you need for these concerns.

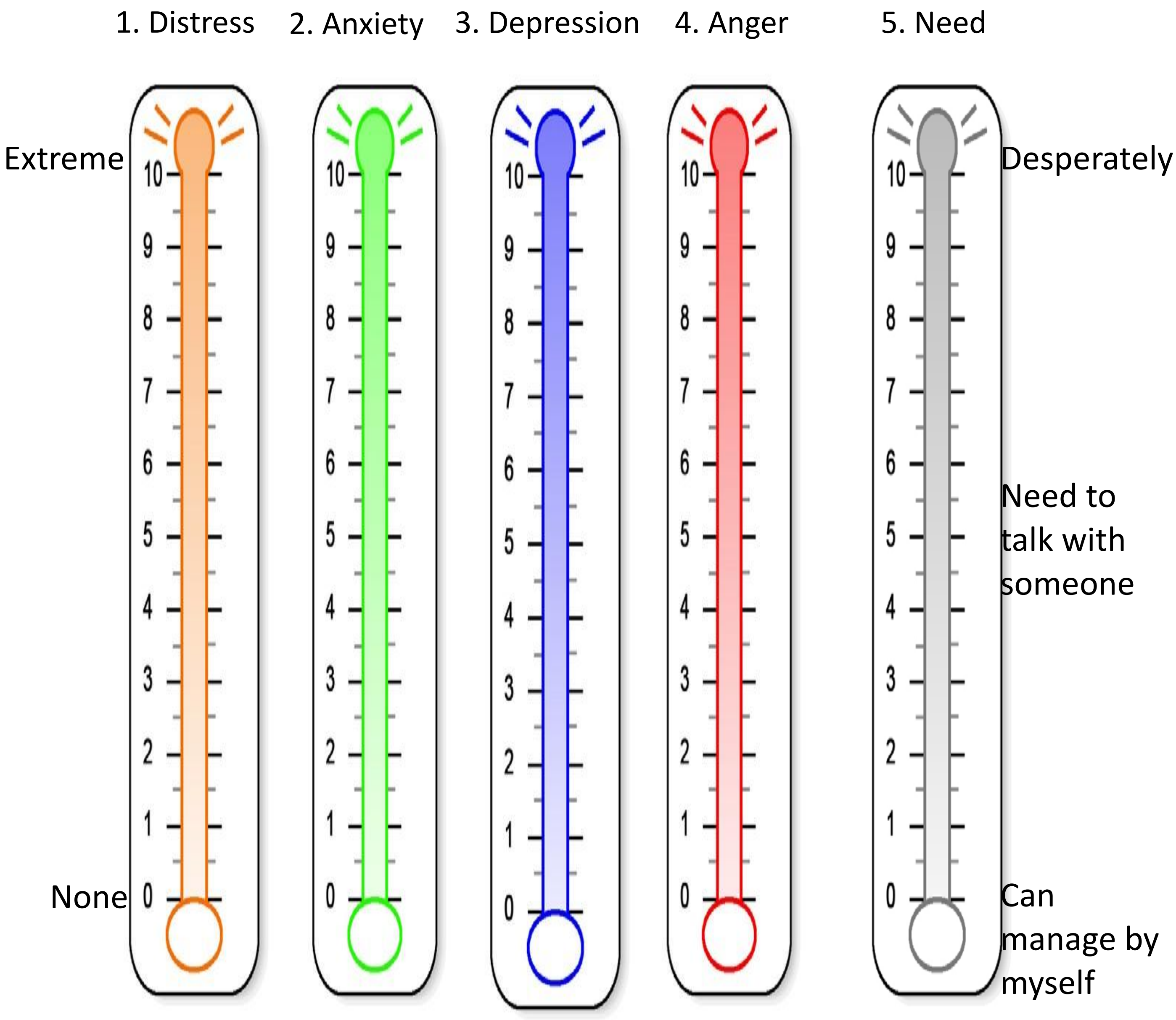

Q.2. *Please indicate if any of the following has been a problem for you in the past week including today. Be sure to fill in* **YES** *or* **NO** *for each.*

Please fill in circles like this: ● Not like this: ⊗ or ✓

Practical problems

YES	NO	
O	O	Child care
O	O	Housing
O	O	Insurance/ financial
O	O	Transportation
O	O	Work/ school

Family problems

YES	NO	
O	O	Dealing with children
O	O	Dealing with partner
O	O	Dealing with close friend/ relative

Emotional problems

YES	NO	
O	O	Depression
O	O	Fears
O	O	Nervousness
O	O	Sadness
O	O	Worry
O	O	Loss of interest in usual activities

Spiritual/religious concerns

YES	NO	
O	O	Any spiritual /religious concerns

Physical problems

YES	NO	
O	O	Appearance
O	O	Bathing/dressing
O	O	Breathing
O	O	Changes in urination
O	O	Constipation
O	O	Diarrhoea
O	O	Eating
O	O	Fatigue
O	O	Feeling swollen
O	O	Fevers
O	O	Getting around
O	O	Indigestion
O	O	Memory/ concentration
O	O	Mouth sores
O	O	Nausea
O	O	Nose dry/ congested
O	O	Pain
O	O	Sexual
O	O	Skin dry/ itchy
O	O	Sleep
O	O	Tingling in hands/ feet

Other problems

Q.3. *This scale consists of a number of words that describe different feelings and emotions. Read each item and then fill in the circle from 1 to 5 to indicate to what extent you have felt this way during the past week.*

Please fill in circles like this: ● Not like this: ⊗ or ✓

1	2	3	4	5
Very slightly or not at all	A little	Moderately	Quite a bit	Extremely

1	2	3	4	5	
O	O	O	O	O	1. Interested
O	O	O	O	O	2. Distressed
O	O	O	O	O	3. Excited
O	O	O	O	O	4. Upset
O	O	O	O	O	5. Strong
O	O	O	O	O	6. Guilty
O	O	O	O	O	7. Scared
O	O	O	O	O	8. Hostile
O	O	O	O	O	9. Enthusiastic
O	O	O	O	O	10. Proud
O	O	O	O	O	11. Irritable
O	O	O	O	O	12. Alert
O	O	O	O	O	13. Ashamed
O	O	O	O	O	14. Inspired
O	O	O	O	O	15. Nervous
O	O	O	O	O	16. Determined
O	O	O	O	O	17. Attentive
O	O	O	O	O	18. Jittery
O	O	O	O	O	19. Active
O	O	O	O	O	20. Afraid

Q.4. *Below are some events renal patients have said made them feel distressed. Please indicate whether any of the events listed have caused you distress in the last week, and in the last 2 months.*

Please fill circles like this: ●   Not like this: ⊗ or ✓

	**In the last week**	**In the last two months**
Being diagnosed with kidney failure	O	O
Being given a poor prognosis	O	O
Making a decision about choice of dialysis treatment	O	O
Adjusting to being on dialysis	O	O
Having dietary restrictions/change in dietary restrictions	O	O
Adjusting to having a transplant	O	O
Death of another renal patient	O	O
A failing transplant	O	O
Crisis in home/work life	O	O
Change to a different type of dialysis	O	O
Problems with dialysis e.g. fistula failure, difficulties with my line, machine problems	O	O
Decision to stop being on dialysis	O	O
Loss of employment/ fear of loss of employment	O	O

Please write in any other events that have caused you distress

**In the last week**	**In the last two months**

The following questions are about support.

Q.5. *How important is it that renal staff provide support for patients when they feel distressed? (Please fill in the circle ● that best describes how important this is to you)* **Please fill circle like this: ●** **Not like this: ⊗ or ✓**

**Not at all important**										**Extremely important**
**0**	**1**	**2**	**3**	**4**	**5**	**6**	**7**	**8**	**9**	**10**
O	O	O	O	O	O	O	O	O	O	O

Q.6. *From your own experience, how satisfied are you with the support you get from renal staff when you feel distressed? (Please fill in the circle ● that best describes how satisfied you are)*

**Not at all satisfied**										**Extremely satisfied**
**0**	**1**	**2**	**3**	**4**	**5**	**6**	**7**	**8**	**9**	**10**
O	O	O	O	O	O	O	O	O	O	O

Q.7. *Below are some things that other patients have said about the support they get from renal staff. For each statement, please mark ●how strongly you agree or disagree.*

	Strongly disagree 0	1	2	3	4	5	6	7	8	9	Strongly agree 10
I can discuss any worries or fears with renal staff	O	O	O	O	O	O	O	O	O	O	O
Sometimes I feel like I'm treated as a set of kidney disease symptoms rather than a whole person	O	O	O	O	O	O	O	O	O	O	O
Renal staff tell me about different types of support available, such as talking with other patients	O	O	O	O	O	O	O	O	O	O	O
The renal staff do not have time to listen	O	O	O	O	O	O	O	O	O	O	O
I feel the renal staff really care	O	O	O	O	O	O	O	O	O	O	O
I don't need the support of renal staff as I get good support from family/friends	O	O	O	O	O	O	O	O	O	O	O
It's very comforting the way renal staff treat me	O	O	O	O	O	O	O	O	O	O	O
I'd like to talk to one of the renal staff, but I don't know who to talk to	O	O	O	O	O	O	O	O	O	O	O

Q.8. *Thinking now about how you have been coping with your illness and treatment, please mark ● how strongly you agree or disagree with the following statements.*

	Strongly disagree 0	1	2	3	4	5	6	7	8	9	Strongly agree 10
I have no problems or difficulties coping with my illness	O	O	O	O	O	O	O	O	O	O	O
I worry that my condition will get worse	O	O	O	O	O	O	O	O	O	O	O
I manage to keep any bad feelings about my illness and treatment under control	O	O	O	O	O	O	O	O	O	O	O
I have difficulty accepting my illness	O	O	O	O	O	O	O	O	O	O	O
I try to think positively about my illness and treatment	O	O	O	O	O	O	O	O	O	O	O
I feel nervous about what will happen in the future	O	O	O	O	O	O	O	O	O	O	O
I'm satisfied with how I'm coping with my illness and treatment	O	O	O	O	O	O	O	O	O	O	O
I worry I might become more distressed in the future	O	O	O	O	O	O	O	O	O	O	O
I feel confident I can handle anything upsetting about my illness and treatment	O	O	O	O	O	O	O	O	O	O	O

Q.9. *Below are listed different types of support that some renal units provide for patients. For each type of support, please mark ● how **helpful** you think you would find this support.*

	Not at all helpful 0	1	2	3	4	5	6	7	8	9	Extremely helpful 10
Time to discuss emotional feelings during nurse-run clinics	O	O	O	O	O	O	O	O	O	O	O
Time to discuss emotional feelings during consultations with a doctor	O	O	O	O	O	O	O	O	O	O	O
Training in how to handle any negative thoughts and feelings	O	O	O	O	O	O	O	O	O	O	O
Talking about my distress with a psychologist/counsellor who knows about renal disease	O	O	O	O	O	O	O	O	O	O	O
A friendly one-to-one chat with another renal patient organised by my renal unit	O	O	O	O	O	O	O	O	O	O	O
An online training course in how to manage my feelings and moods	O	O	O	O	O	O	O	O	O	O	O
Marking on a sheet given to me by the staff, the issues I want to discuss in my clinic/consultation	O	O	O	O	O	O	O	O	O	O	O
A personal physical activity programme designed by renal staff	O	O	O	O	O	O	O	O	O	O	O
Joining an art or drawing group arranged by my renal unit	O	O	O	O	O	O	O	O	O	O	O
While I'm dialysing, talking with a nurse about my distress	O	O	O	O	O	O	O	O	O	O	O
An online support group with other renal patients	O	O	O	O	O	O	O	O	O	O	O

Please write in any other types of support that you would like your renal unit to provide.

Now some questions about you.

If you are helping someone to complete this questionnaire, please make sure this information is the patient's, not your own.

Q.10. ***Your age***: *in what year were you born? (Please write in)*

____

Q.11. *Are you male or female?* (*Please mark* ●)

Male O

Female O

Q.12. *How long has it been since you were diagnosed with end-stage renal disease (chronic kidney disease stage 5)? (please mark ●)*

Less than 6 months ago O

Between 6 months 12 months ago O

1+ year to 2 years ago O

2+ years to 3 years ago O

3+ years to 5 years ago O

5+ years to 10 years ago O

10+ years to 15 years ago O

More than 15 years ago O

Q.13. *At what stage is your treatment for end-stage renal disease? (please mark ●)*

Not yet started renal replacement treatment	O
On dialysis for less than 2 years, and not on the transplant waiting list	O
On dialysis for 2 or more years, and not on the transplant list	O
On dialysis and on the transplant list	O
With a transplant	O
On dialysis after a failed transplant	O
On conservative management/ chosen not to be on dialysis	O

Other (please write in)____

If you are on dialysis, please answer Q.14.
If you are not on dialysis, please go to Q.15.

Q.14. *What type of dialysis are you on? (please mark ●)*

Haemodialysis (HD) in a hospital	O
Haemodialysis (HD) in a satellite unit	O
Home haemodialysis (HHD)	O
Continuous ambulatory peritoneal dialysis (CAPD)	O
Automated peritoneal dialysis (APD)	O

Q.15. *What is your ethnic group? Please mark ●the one option below that best describes your ethnic group or background.*

White

- English/Welsh/Scottish/ Northern Irish/British O
- Irish O
- Gypsy or Irish Traveller O
- Any other White background O

Mixed/Multiple ethnic groups

- White and Black Caribbean O
- White and Black African O
- White and Asian O
- Any other Mixed/Multiple ethnic background O

Asian/Asian British

- Indian O
- Pakistani O
- Bangladeshi O
- Chinese O
- Any other Asian background O

Black/African/Caribbean/ Black British

- African O
- Caribbean O
- Any other Black/ African/ Caribbean background O

Other ethnic group

- Arab O
- Any other ethnic group O

Q.16. *What are your current living arrangements? Please mark ●the one option below that best describes your living arrangements.*

I live on my own O

I live with a partner or family member(s) O

Other (please describe)

____

Q.17. *Has anyone helped you fill-in this questionnaire? Please mark* ● for either yes or no

Yes O

No O

Q.18. *Finally, would you be willing to help us with further research on these issues, by taking part in a 35-45 minute telephone or face-to-face interview, with a researcher from the University of Birmingham, sometime over the next two months? (We can provide over-the-phone interpreting for anyone that would like this).*

Please mark ● *if willing to help us with further research by taking part in an interview*

O Yes, I would be willing to take part in a 35-45 minute interview

Please provide your contact details:

Phone number/Email ____

UNIVERSITY OF BIRMINGHAM

Other comments

If there is anything else you would like to tell us about your emotional needs as a renal patient, please do so here.

To send us your views, please send your completed questionnaire to the University of Birmingham in the FREEPOST envelope provided. You do not need to add any postage.

THANK YOU VERY MUCH FOR YOUR HELP.

For university use

Number	Site	Stage
