# Supplementary material for: Integrating emotional and psychological support into the end-stage renal disease pathway: a protocol for mixed methods research to identify patients’ lower-level support needs and how these can most effectively be addressed
Source: BMC Nephrol. 2016 Aug 2;17:111. doi: 10.1186/s12882-016-0327-2 (PMC4971672; doi:10.1186/s12882-016-0327-2)
Supplement: Additional file 2: — Staff questionnaire. Questionnaire developed for Study 2. (PDF 2781 kb) [file 12882_2016_327_MOESM2_ESM.pdf]

(https://s3-eu-west-1.amazonaws.com/aws.smartsurvey.co.uk/_files/cabinet/79504/47879_university-of-birmingham-3.png)

Supporting the emotional needs of end-stage renal disease patients

0%

We are inviting you to take part in this study on identifying and responding to the needs of patients with end-stage renal disease (ESRD) who are in distress. If you agree to take part, please fill-in this questionnaire. We encourage you to answer all the questions, but if there is a question you do not want to answer, you can choose to leave it blank, and go onto the next question. The answers you provide will be kept confidential.

The following questions all relate to *distress* in patients with ESRD. By distress we mean the lower-level emotional and psychological difficulties experienced by patients: the unpleasant feelings or emotions that may interfere with patients' ability to cope with kidney failure, its physical symptoms and its treatment. *Distress* covers a wide range of feelings including anger, frustration, sadness, fear, depression, guilt and anxiety.

If you have any problems completing this survey, or any questions, please contact Francesca Taylor, University of Birmingham (f.taylor@bham.ac.uk; 07775 531674).

These first questions are about ESRD patients in distress

1. What proportion of ESRD patients in your Renal Unit do you think are in distress? (Please mark)

Under 10%	10-20%	21-30%	31-40%	41-50%	51-60%	61-70%	71-80%	Over 80%	Don't know
○	○	○	○	○	○	○	○	○	○

2. Do you feel it is beneficial or not for ESRD patients' overall health that they receive support for their distress from their Renal Unit? (Please mark)

Not at all beneficial 0	1	2	3	4	5	6	7	8	9	Very beneficial 10
○	○	○	○	○	○	○	○	○	○	○

3. How satisfied are you with the support your Renal Unit provides for ESRD patients in distress? (Please mark)

Not at all satisfied 0	1	2	3	4	5	6	7	8	9	Very satisfied 10
○	○	○	○	○	○	○	○	○	○	○

Next Page

Powered by **SmartSurvey (https://www.smartsurvey.co.uk)**

(https://s3-eu-west-1.amazonaws.com/aws.smartsurvey.co.uk/_files/cabinet/79504/47879_university-of-birmingham-3.png)

Supporting the emotional needs of end-stage renal disease patients

11%

The following questions are about identifying and responding to ESRD patients in distress

4. Below is a list of statements that other renal staff have made about being responsible for identifying and responding to the needs of ESRD patients in distress. How much do you agree or disagree with each statement? (Please mark)

	Strongly disagree 0	1	2	3	4	5	6	7	8	9	Strongly agree 10
It's an important part of my work.											
It's the responsibility of renal staff other than me											
It's the responsibility of other professions, such as GPs, counsellors or voluntary organisations											
It's included in my job description											

	Strongly disagree 0	1	2	3	4	5	6	7	8	9	Strongly agree 10
I've not been trained for this role	○	○	○	○	○	○	○	○	○	○	○
I find it a fulfilling role	○	○	○	○	○	○	○	○	○	○	○
There's little point in taking on this responsibility as there's no reward in salary/promotion	○	○	○	○	○	○	○	○	○	○	○
I recognise the benefits for my patients if I take on this role	○	○	○	○	○	○	○	○	○	○	○

Previous Page Next Page

Powered by **SmartSurvey (https://www.smartsurvey.co.uk)**

(https://s3-eu-west-1.amazonaws.com/aws.smartsurvey.co.uk/_files/cabinet/79504/47879_university-of-birmingham-3.png)

Supporting the emotional needs of end-stage renal disease patients

22%

5. Below is a list of statements that other renal staff have made about how well they are able to identify and respond to the needs of ESRD patients in distress. How much do you agree or disagree with each statement? (Please mark)

	Strongly disagree 0	1	2	3	4	5	6	7	8	9	Strongly agree 10
I find it difficult to know when a patient is distressed	○	○	○	○	○	○	○	○	○	○	○
I need more training to help me	○	○	○	○	○	○	○	○	○	○	○
I have no problems or difficulties that prevent me doing this well	○	○	○	○	○	○	○	○	○	○	○
I don't have the time	○	○	○	○	○	○	○	○	○	○	○
There's no where to refer patients for support if you identify they are distressed	○	○	○	○	○	○	○	○	○	○	○
I do this effectively	○	○	○	○	○	○	○	○	○	○	○

	Strongly disagree 0	1	2	3	4	5	6	7	8	9	Strongly agree 10
I find it difficult to do because it is too emotionally demanding for me	◯	◯	◯	◯	◯	◯	◯	◯	◯	◯	◯
I don't have the right skills	◯	◯	◯	◯	◯	◯	◯	◯	◯	◯	◯
I'm given good support by my Renal Unit to help me to do this	◯	◯	◯	◯	◯	◯	◯	◯	◯	◯	◯

Previous Page Next Page

Powered by **SmartSurvey** (https://www.smartsurvey.co.uk)

(https://s3-eu-west-1.amazonaws.com/aws.smartsurvey.co.uk/_files/cabinet/79504/47879_university-of-birmingham-3.png)

Supporting the emotional needs of end-stage renal disease patients

33%

Please answer Qs 6 and 7 if it is part of your role to identify and respond to the needs of ESRD patients in distress. If it is not part of your role, please go to Q 8 on the next page

6. Thinking now about factors that would help you identify and respond to the needs of ESRD patients in distress, please indicate how helpful each of the things listed below would be for you. (Please mark)

	Not very helpful 0	1	2	3	4	5	6	7	8	9	Very helpful 10
Longer time slots with patients											
A screening tool to identify patients who need support											
A specialist nurse I can refer patients to											

	Not very helpful 0	1	2	3	4	5	6	7	8	9	Very helpful 10
Skills training on how best to support patients	○	○	○	○	○	○	○	○	○	○	○
Recognition in terms of pay/promotion	○	○	○	○	○	○	○	○	○	○	○
Interventions available that I can suggest to patients e.g. peer support	○	○	○	○	○	○	○	○	○	○	○
Access to a renal psychologist for patients	○	○	○	○	○	○	○	○	○	○	○
Patients being able to see the same nurse at each clinic	○	○	○	○	○	○	○	○	○	○	○
Knowing where to signpost patients for help	○	○	○	○	○	○	○	○	○	○	○
Emotional support for staff who are providing emotional support to patients	○	○	○	○	○	○	○	○	○	○	○
Knowing which patients to particularly target and when	○	○	○	○	○	○	○	○	○	○	○

Please write in any other factors, not mentioned above, that you think would be helpful

7. What 3 or 4 things would most help your Renal Unit better identify and respond to the needs of ESRD patients in distress? (Please write in)

(https://s3-eu-west-1.amazonaws.com/aws.smartsurvey.co.uk/_files/cabinet/79504/47879_university-of-birmingham-3.png)

Supporting the emotional needs of end-stage renal disease patients

44%

Now some questions about yourself

8. Your age: *in what year were you born?* (Please write in)

9. Are you male or female?

- Male
- Female

10. What is your mother's date of birth? (Please write in dd/mm/yyyy)

(This will help us with cross-checking if we repeat the questionnaire)

Previous Page Next Page

Powered by **SmartSurvey** (https://www.smartsurvey.co.uk)

(https://s3-eu-west-1.amazonaws.com/aws.smartsurvey.co.uk/_files/cabinet/79504/47879_university-of-birmingham-3.png)

Supporting the emotional needs of end-stage renal disease patients

56%

11. Which of the following best describes your current role?

- Specialist nurse
- Ward nurse
- Dialysis nurse
- Healthcare assistant
- Consultant
- Registrar
- Dietician
- Technician
- Psychologist/ Social worker/ Welfare advisor/ Counsellor
- Renal manager
- Other

Other (please write in)

12. Which Hospital Trust do you currently work for?

- The Royal Wolverhampton NHS Trust
- Heart of England NHS Trust
- Other

Other (please write in)

13. How long have you been working in your current role at this Hospital Trust?

- Less than 6 months
- 6 – 12 months
- 12+ months – 2 years
- 2+ years – 3 years
- 3+ years – 5 years
- 5+ years – 10 years
- 10+ years – 20 years
- 20+ years – 30 years
- More than 30 years

Previous Page   Next Page

Powered by SmartSurvey (https://www.smartsurvey.co.uk)

(https://s3-eu-west-1.amazonaws.com/aws.smartsurvey.co.uk/_files/cabinet/79504/47879_university-of-birmingham-3.png)

Supporting the emotional needs of end-stage renal disease patients

67%

14. How often do you have contact with ESRD patients?

- Every working day
- Most working days
- 3-4 days a week
- Weekly
- Every 2-3 weeks
- Monthly
- Less often than monthly

15. How long ago did you qualify for your current role?

- Under 6 months
- 7 – 12 months
- 12+ months – 2 years
- 2+ years – 3 years
- 3+ years – 5 years
- 5+ years – 10 years
- 10+ years – 20 years
- 20+ years – 30 years
- 30+ years

16. Have you received training in how to identify and respond to the needs of ESRD patients in distress?

	Yes	No
As part of my initial professional training	◯	◯
As part of the training for my current role	◯	◯
While in my current role	◯	◯
During the last 3 years	◯	◯

Previous Page Next Page

Powered by **SmartSurvey (https://www.smartsurvey.co.uk)**

(https://s3-eu-west-1.amazonaws.com/aws.smartsurvey.co.uk/_files
/cabinet/79504/47879_university-of-birmingham-3.png)

Supporting the emotional needs of end-stage renal disease patients

78%

17. Finally, would you be willing to help us with further research on these issues, by taking part in a 30-minute telephone or face-to-face interview, sometime over the next two months?

- Yes, I would be willing to participate in a 30-minute telephone/face-to-face interview

Please provide your contact details - Email/ telephone number

Other comments

If there is anything else about supporting the emotional needs of ESRD patients with distress that you think is important and you want to tell as about, please write in below

Previous Page | Next Page

Survey Preview You are currently previewing this survey, no data will be saved. Jump to: Page 9

(https://s3-eu-west-1.amazonaws.com/aws.smartsurvey.co.uk/_files/cabinet/79504/47879_university-of-birmingham-3.png)

Supporting the emotional needs of end-stage renal disease patients

89%

If you agree for your responses to be used anonymously as part of the study undertaken by the University of Birmingham, please click 'Finish survey' to submit.

Thank you for your time and assistance.

Previous Page | Finish Survey

Powered by **SmartSurvey** (https://www.smartsurvey.co.uk)
